# The Infant Brain

**Published:** 1891-06

**Authors:** 


### THE INFANT BRAIN.

The brain of a child is proportionately much larger than an adult's, but of much softer consistency, and its convolutions are not complete until the seventh year. This is one of the reasons why early study is dangerous. The child's heart beats much more rapidly than that of an adult, and the growth of the heart, instead of being regular, like the growth of the body as a whole, is accomplished by fits and starts. The more rapid action of the heart renders the child peculiarly liable to fever, and the liability is further increased by his weaker vital resistance. Hence childhood is the special season for scarlet fever, measles, whooping-cough, and other similar complaints.

The irregularity of the heart's growth may give rise to disturbances of that organ of a seemingly dangerous character, but with proper care they will pass away as the heart attains its full development. Such proper care includes ample nourishment, sufficient sleep, and the avoidance of special strain. All children are curious, all children love approbation, and all have a certain sense of justice. They are naturally active, and when a child mopes it is in bad physical condition. When a child is fidgety, give him something to do. The surplus nervous force must work off. If you find the right way, nothing will so much save the clashing of will power between the mother and the child.

Nature is constantly prompting the child to do something, and we are trying to keep him from it. Give him something to play with—block games or something that will amuse him. In this manner you can entirely do away with the wrong use of the hands. One of the great truths of the kindergarten is, that if we will control the right tendencies, the wrong tendencies in the child will die out. Cultivate the right kind of characteristics. The child finally reaches the period when he realizes the appalling power of destruction. He destroys every thing he gets his hands on. It is a natural characteristic. It is that instinct of the child that makes him want to push himself into the work. This instinct is of an investigating nature. When it takes the form of destruction it is bad, of course, and creates a certain spendthrift tendency. But the child must investigate. Give him toys he can take apart, and show him how it is done. Give him a horse that can be hitched and unhitched to the cart at his pleasure. This is one of the principles of Froebel's theory.

The season of rapid growth and development, say between the ages of ten and twenty, needs particular attention. Nature is then at work, as it never will be again, in building up the tissues and developing the nervous sensibilities. This is the period which makes the largest demands for an outdoor life, for pure air, sunlight, active exercise, abundance of nutritous food, a vigorous digestive tract, a ready assimilation, and an active elimination of waste. It is the period of study and ambition, as well as of a wisdom that thinks itself wiser than it is. The increasing mental activity needs to be regulated by experienced teachers and considerate mothers, lest the brain be worked at the expense of other organs and tissues. Duller minds should not be forced to keep step with those which are naturally more active, and the influences of the home and the schoolroom should be tranquilising and adapted to evoke the kindlier feelings. Fretful parents and scolding teachers may do a life long injury during this susceptible period. It is a period when neither study nor night excitements should interfere with sleep ; when bad novels do their worst work ; when parents need to know what their children read, and to be their confidential counsellor in all delicate matters ; when the use of tobacco is specially perilous, almost surely giving rise to affections of the heart, and when spirituous liquors and all opiates are peculiarly pernicious.

